# Articular mobilization promotes improvement in functional and inflammatory parameters in a gouty arthritis model

**DOI:** 10.31744/einstein_journal/2023AO0465

**Published:** 2023-10-18

**Authors:** Iranilda Moha Hoss, Lilian de Araujo Pradal, Taciane Stein da Silva Leal, Gladson Ricardo Flor Bertolini, Rose Meire Costa, Lucinéia de Fátima Chasko Ribeiro

**Affiliations:** 1 Universidade Estadual do Oeste do Paraná Cascavel PR Brazil Universidade Estadual do Oeste do Paraná, Cascavel, PR, Brazil.

**Keywords:** Arthritis, gouty, Inflammation, Leukocytes, Synovial fluid, Edema, Models, theoretical, Rats, Wistar

## Abstract

**Objective:**

Gouty arthritis is characterized by painful inflammation due to the deposition of monosodium urate crystals in joint tissues. Despite available treatments, many patients experience ineffective management and adverse effects. This study evaluated a manual therapy protocol involving passive joint mobilization at the peak of inflammation in a gouty arthritis model using functional and inflammatory parameters.

**Methods:**

Twenty male Wistar rats, 12 weeks old, were divided into two groups (n=10 each): Gouty Arthritis and Control Groups, which were further subdivided into treated and untreated groups (n=5 each). The Gouty Arthritis Group received intraarticular knee injection of 50µL of monosodium urate crystals, while the Control Group received 50µL of phosphate buffered saline. The treatment involved a 9-minutes session of grade III joint mobilization (according to Maitland). Nociception, grip strength, and edema were evaluated before induction (EV_0_), 7 hours after assessment (EV_1_), immediately after treatment (EV_2_), and 1 hour after treatment (EV_3_). The animals were euthanized, and synovial fluid was collected to analyze leukocyte migration.

**Results:**

The model mimicked the signs of the Gouty Arthritis Group, with a decrease in the threshold of nociception and strength and an increase in edema and leukocyte count. The mobilization protocol significantly increased the nociceptive threshold and grip strength and reduced edema; however, it did not reverse the increase in leukocyte count.

**Conclusion:**

Our results suggest that mobilization promotes analgesia and may modulate the inflammatory process owing to reduced edema and subtle attenuation of cell migration, which contributes to strength gain.

## In Brief

Hoss et al. reproduced a model simulating the characteristics of the gouty arthritis group, with a decrease in the threshold of nociception and strength and an increase in edema and leukocyte count. The mobilization protocol significantly increased the nociceptive threshold and grip strength and reduced edema; however, it did not reverse the increase in leukocyte count. Our results suggest that mobilization promoted analgesia and may modulate the inflammatory process through reduced edema and subtle attenuation of cell migration, which contributed to the strength gain.

## Highlights

The predominance of neutrophils was observed in an acute model of gout in mice, where neutrophils were mobilized and gradually replaced by monocytes/macrophages, with attenuation of the influence of leukocyte infiltration in the synovial fluid.

Mobilization promotes analgesia and modulates the inflammatory process due to reduced edema and subtle attenuation of cell migration.

At the inflammatory peak in a gouty arthritis model, manual therapy and passive joint mobilization decrease nociception and edema in functional and inflammatory parameters.

## Visual Abstract

**Figure:**
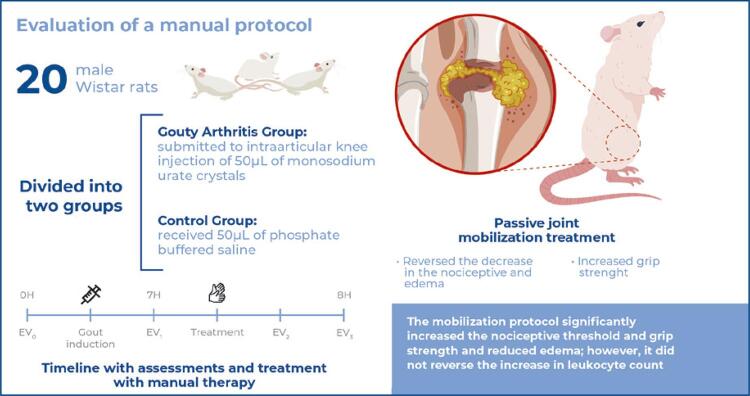

## INTRODUCTION

Gouty arthritis (GA), resulting from hyperuricemia, is a metabolic disorder involving uric acid^(1)^ that promotes the formation and deposition of monosodium urate (MSU) crystals in the periarticular^(2)^ and intraarticular tissues.^(3)^ These crystals activate innate immune system components and generate an intense local inflammatory response.^(1,4,5)^

The prevalence of GA is estimated to be 2% of the world’s population, with high costs related to treatment maintenance, absenteeism, and presenteeism.^(6)^ This disease is prevalent in men aged >40 years. Its incidence is associated with increased longevity, lifestyle changes,^(4)^ diet,^(7)^ and increased prevalence of metabolic syndrome.^(8,9)^

The acute phase is characterized by sudden and self-limiting signs^(4,10)^ and classic monoarticular involvement.^(7)^ Approximately 92.85% of patients with GA experience knee outbreaks^(11)^ with severe pain,^(3)^ erythema,^(7)^ and edema.^(3,12)^ The inflammatory peak is reached in 6-12 hours when leukocyte migration occurs, with a predominance of polymorphonuclear cells.^(12-14)^

The use of drugs is recommended as a treatment strategy to reduce inflammatory responses. These include non-steroidal anti-inflammatory drugs, colchicine,^(15)^ and antiuricosuric drugs, to maintain uric acid levels below the saturation point (<6mg/dL).^(16)^ This prevents the formation of crystals and aids in the dissolution of preexisting crystals.^(17)^ Despite the availability and efficacy of these drugs, a significant number of patients (approximately 69%)^(18)^ cannot achieve GA control due to adverse gastrointestinal and renal effects. Some patients do not adhere to the treatment properly or refuse pharmacological treatment.^(16)^ Therefore, ineffective medication management can result in disease progression and chronicity.^(18)^

Therefore, there is a need for affordable alternative treatments with fewer adverse effects to minimize pain and inflammatory edema, thereby contributing to the improvement of joint function. Joint mobilization is a manual therapy technique that consists of passive and oscillatory maneuvers^(19,20)^ performed in synovial articulation in patient treatment protocols^(19)^ and experimental animal models^(21)^ within the normal range of motion.^(19,20)^

It has been speculated that the oscillatory maneuvers of passive joint mobilization promote a rapid analgesic effect,^(22)^ possibly activating the descending pain inhibitory pathways in humans^(23,24)^ and increasing mechanical nociception in animals, thereby promoting a neurophysiological effect.^(21,25,26)^ Among the forms of applicability of joint mobilization,^(23,26)^ grade III, according to Maitland,^(20)^ is a mobilization performed with a wide range of motion.^(19)^ Despite the limited evidence in the experimental area, it suggests that mechanical action stimulates excitable cells and activates endogenous pain inhibitory pathways,^(21)^ promoting primary^(25)^ and secondary mechanical hypoalgesia.^(21)^

However, till date, the effect of mobilization in an acute model of GA has not been directly known, and there is limited scientific evidence on the modulation of the inflammatory response to mobilization, especially at the inflammatory peak of the disease. Mobilization can promote analgesia, affect fluid dynamics, stimulate synovial fluid production, and support the elimination of byproducts of the inflammatory process, thus reducing edema^(20)^ and enhancing joint function.^(26)^

## OBJECTIVE

To analyze the effects of grade III passive joint mobilization during the inflammatory peak on functional and inflammatory parameters in an experimental model of gouty arthritis.

## METHODS

### Study type and animals

An experimental, randomized, non-blinded study was conducted, which involved using 20 male *Wistar* rats aged 12 weeks and weighing 300g. The animals were kept under controlled conditions of temperature (22±1 °C) and light (12:12 hours light/dark cycle) and received water and feed *ad libitum*. All experiments were performed following the ethical guidelines defined by the International Association for the Study of Pain and approved by the Ethics Committee for the Use of Animals of *Universidade Estadual do Oeste do Paraná* # 19-20.

The animals were randomly separated and divided into four groups (n=5 each). The control (CON) group received an intraarticular injection of phosphate-buffered saline solution (PBS) (50µL) and did not undergo any form of treatment. The GA Group received the MSU intraarticular injection (50µL; 1.25mg) and did not receive any treatment. The control mobilization (CONM) group received an intraarticular injection of PBS (50µL) and was treated with the passive articular mobilization protocol. The GA mobilization (GAM) group received MSU intraarticular injection (50µL; 1.25mg) and was treated with the same protocol as CONM.

### Experimental model of gouty arthritis

The GA model, as described by Coderre et al.^(27)^ involves the intraarticular injection of MSU crystals into the knee joints of animals. The crystals were produced at the Structural and Functional Biology Laboratory, as described by Tavares et al.,^(14)^ according to the following protocol: 4g of uric acid was dissolved in 800mL of PBS, and the pH was adjusted to 8.9. This dilution was maintained in an oven at 50^o^C until total evaporation. The product consisted of MSU crystals suspended in PBS (pH 7.4).

To induce experimental GA, the animals in the GA and GAM Groups were carefully immobilized with a flannel, leaving only the lower third of the abdomen and pelvic limbs free, and placed in the supine position with the right pelvic member flexed. Next, the right knee was trichotomized, the local area was disinfected with iodized alcohol solution, and intraarticular injection of MSU crystals was administered (1.25mg of MSU suspended in 50µL of PBS). The animals in the CON and CONM Groups underwent the same procedure but received 50µL of PBS.

### Functional assessments

Prior to the experiment, the animals were acclimatized and trained in functional tests (three sessions interspersed for 7 days). All tests were performed by the same researcher, and all experimental groups were acclimated.

The following parameters were verified during the evaluation: nociceptive threshold, grip strength, and joint edema. The nociceptive threshold was assessed using a digital Von Frey analgesiometer (Insight^®^, Ribeirão Preto, São Paulo), with the values expressed in grams (g), as described by Möller et al.^(28)^ The grip strength was assessed using a grip strength meter (Insight^®^, Ribeirão Preto, São Paulo), as described by Bertelli et al.^(29)^ with values expressed in grams (g). Edema was assessed by measuring the femoral-tibial joint diameter (medial-lateral axis) using a manual pachymeter, and the results were expressed in millimeters (mm) according to Bressan et al.^(30)^ Three measurements were performed for all functional parameters, and the average value of each evaluation was used.

**Figure 1:**
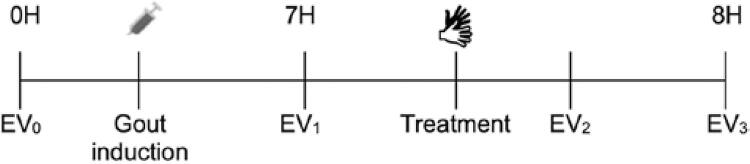
Timeline with assessments and treatment with manual therapy

A baseline evaluation (EV_0_) was performed before GA induction. After 7 hours, the first functional evaluation (EV_1_) was performed, and the animals were immediately subjected to a new evaluation (EV_2_). One hour after EV_2_, a final evaluation (EV_3_) was performed, as shown in figure 1.

### Treatment protocol with joint mobilization

Animals in the groups (CONM and GAM) received manual therapy with a single session of grade III passive joint mobilization on the right knee, according to Maitland. This technique was performed with animals in the supine position using a flannel. The researcher used their first and second fingers to create tweezers to stabilize the rodent’s femur and, with the dominant hand, performed rhythmic tibial flexion and extension movements within the normal range of motion but with great amplitude, thereby performing the grade III joint mobilization, according to Maitland and as described in animals by Sluka et al.^(25)^ The protocol was performed by the same researcher, with three repetitions of 3 minutes each, with 30 seconds of rest between each mobilization, for a total of 9 minutes of treatment, as described by Sluka et al.^(21)^

### Leukocyte migration

**Figure 2:**
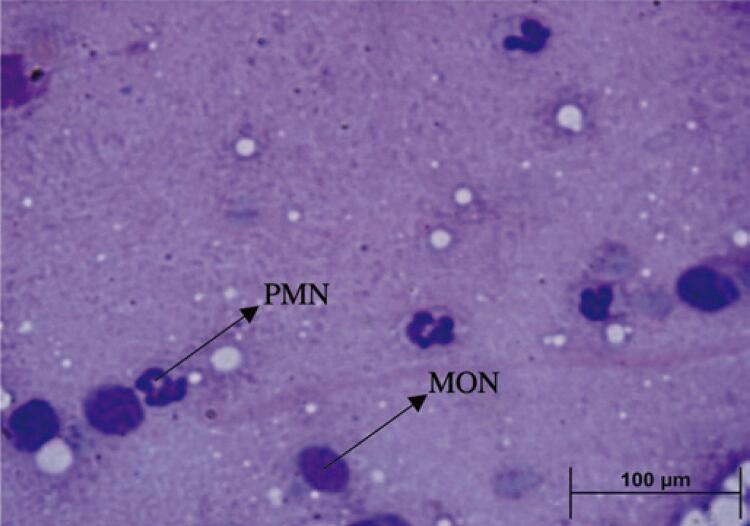
Photomicrograph of the smear slide stained in May-Grunwald and Giemsa, in 100x magnification. Mononuclear and polymorphonuclear cells

The animals were anesthetized with an intraperitoneal injection of 240mg/kg ketamine hydrochloride and 45mg/kg xylazine and then euthanized by anesthetic overdose. After the state of consciousness of the animal was verified by observing the absence of a motor response to the clamping of the tail, 5μL of synovial fluid from the right knee joint was collected to prepare smears for staining with May-Grunwald and Giemsa. Subsequently, the percentages of mononuclear and polymorphonuclear cells (Cells % of MON and PMN) were determined using a light microscope fitted with a 100× objective in a blinded manner. For the total count of leukocyte migration, the joint was washed with an anticoagulant solution composed of 100μL of 0.9% physiological saline and 4μL of EDTA 5% with a pipette. After 20μL of the wash was used, it was diluted in Turk’s liquid, with dilution factor varying between 180 and 480µL depending on the concentration of cells in the synovial fluid, according to Gomes et al.^(31)^ Counting was performed in a Neubauer glass chamber (cells/mm^3^); four quadrants were adopted to measure the number of leukocytes, and the analysis was conducted using a light microscope fitted with a 40× objective, as shown in figure 2.

### Statistical analysis

Statistical analyses were performed using SPSS Statistics version 20.0 software (IBM Corp., Armonk, NY, USA). A mixed generalized linear test and a least significant difference post-hoc test were used to test the functional analyses. A generalized linear test was used to analyze leukocyte migration. Data are expressed as the mean and standard error. A 95% confidence interval was used for all the tests. Statistical significance was set at p<0.05.

## RESULTS

**Figure 3:**
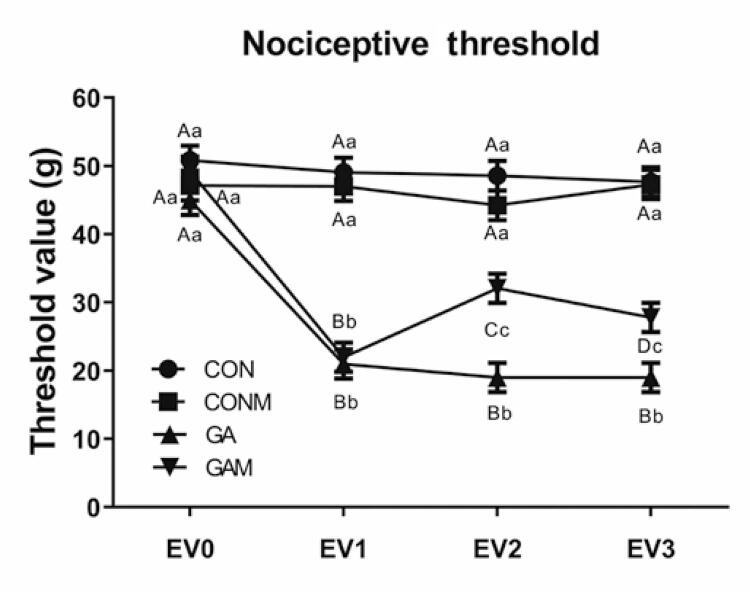
Behavior of the nociception threshold between groups and assessments

The assessment of the nociceptive threshold (Figure 3) showed differences between the groups and assessments, as well as interactions between factors (p<0.001). At baseline, the groups were homogeneous; however, in EV_1_, the two groups that received experimental gout showed a reduction in their thresholds. In EV_2_, the GA Group presented a lower threshold than the three other groups. In contrast, the GAM Group showed a higher threshold than the GA Group but a lower threshold than the other two groups.

Results expressed as mean±standard error. Accepted p<0.05. Equal capital letters represent statistical similarity between the experimental groups. Equal lowercase letters represent statistical similarity between assessments.

CON: Control Group; CONM: Control Group Mobilization; GA: Gouty Arthritis Group; GAM: Gout Group Mobilization.

During the intragroup evaluation, both the control and mobilization groups remained stable throughout the evaluations. The GA Group showed a reduction in thresholds when comparing the baseline assessment with subsequent assessments. Finally, the GAM Group also showed a higher baseline assessment than the subsequent assessments; however, EV_2_ had a higher nociceptive threshold than EV_1_.

**Figure 4:**
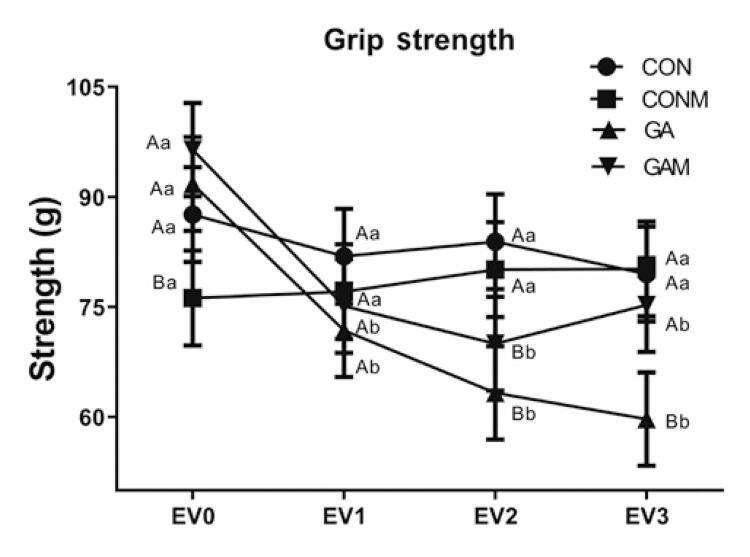
Grip strength behavior between groups and assessments

The analysis of grip strength, as shown in figure 4, revealed statistically significant differences between the groups (p=0.001), assessments (p=0.005), and the interaction of factors (p=0.001). Among the experimental groups, the GA and GAM Groups exhibited a significant decrease in grip strength compared to the CON and CONM Groups (p=0.001).

Results expressed as mean±standard error. Accepted p<0.05. Equal capital letters represent statistical similarity between the experimental groups. Equal lowercase letters represent statistical similarity between assessments.

CON: Control Group; CONM: Control Group Mobilization; GA: Gouty Arthritis Group; GAM: Gout Group Mobilization.

In the intragroup interactions, the CON and CONM Groups showed no significant changes during the evaluations. However, the GA Group exhibited a significant decrease in grip strength in EV_2_, lasting until EV_3_, compared to EV_1_ (p=0.001). In contrast, the GAM Group showed a significant reversal of decreased grip strength in EV_3_ compared to that in EV_1_ (p=0.006).

**Figure 5:**
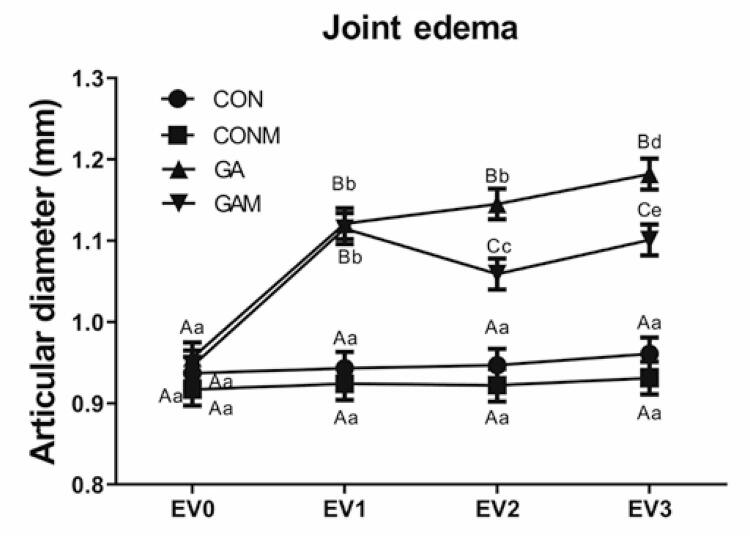
Behavior of inflammatory edema between groups and between assessments

For the analysis of joint edema (Figure 5), statistically significant differences were observed between the experimental groups and assessments, as well as significant interactions of factors (p=0.001). Groups GA and GAM showed a significant increase in joint diameter compared with the CON and CONM Groups (p=0.001). Joint mobilization in the GAM Group significantly reversed the increase in diameter compared with that in the GA Group (p=0.001), an effect that persisted until EV_3_ (p=0.001).

Results expressed as mean±standard error. Accepted p<0.05. Equal capital letters represent statistical similarity between the experimental groups. Equal lowercase letters represent statistical similarity between assessments.

CON: Control Group; CONM: Control Group Mobilization; GA: Gouty Arthritis Group; GAM: Gout Group Mobilization.

In the intragroup analysis, both the CON and CONM Groups remained stable throughout the evaluation. However, the GA Group showed a significant increase in joint diameter when EV_0_ was compared with subsequent evaluations (p=0.001). In the GAM Group, a significant increase in joint diameter was observed when comparing EV_1_ with EV_0_ (p=0.001). However, treatment involving joint mobilization reversed the increase in joint diameter when EV_1_ was compared with EV_2_ and EV_3_ (p=0.001).

**Figure 6:**
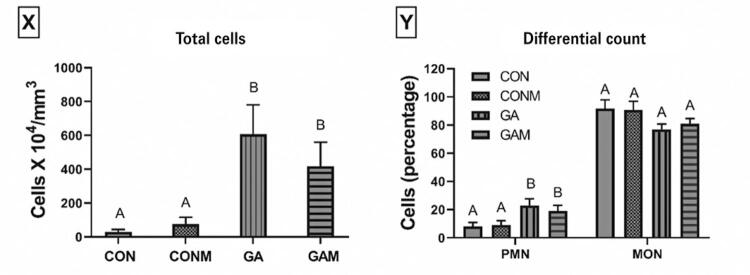
Leukocyte migration in synovial fluid of animals with induction of experimental gouty arthritis

Leukocyte migration analysis (Figure 6X) of the total leukocyte count revealed a statistically significant difference between the groups (p=0.001). The GA and GAM Groups had significantly higher leukocyte counts than the CON and CONM Groups (p=0.001 and 0.003, respectively). Treatment with the joint mobilization protocol did not reverse this increase compared to that in the GA Group (p=0.399).

Results expressed as mean standard error. Accepted p<0.05. Equal capital letters represent statistical similarity between the experimental groups.

X: Total leukocyte count; Y: Percentage of polymorphonuclear (PMN) and mononuclear cells (MON); CON: Control Group; CONM: Control Group Mobilization; GA: Gouty Arthritis Group; GAM: Gout Group Mobilization.

The percentage of polymorphonuclear cells (Figure 6Y) showed a statistically significant difference between the groups (p=0.013). In the GA and GAM Groups, the percentage of cells was significantly higher than that in the CON (p=0.007 and 0.022, respectively) and CONM (p=0.014 and 0.046, respectively) groups. Treatment with mobilization did not significantly reverse the percentage of polymorphonuclear cells compared with that in the GA Group (p=0.525). The percentage of mononuclear cells did not differ significantly between the experimental groups (p=0.088).

## DISCUSSION

This study provided evidence that passive joint mobilization can be beneficial in treating GA during the acute phase of the disease. The MSU crystal-induced gout model showed reduced nociceptive threshold and grip strength, as well as increased joint diameter and leukocyte recruitment during the inflammatory peak, while joint mobilization reversed the effects observed on nociception, grip strength, and inflammatory edema. The presence of intraarticular urate crystals causes leukocyte recruitment, resulting in a local inflammatory response in the presence of pain, edema, and cell migration.^(12-14)^

In the present study, the arthritic groups exhibited a decrease in the nociceptive threshold at the inflammatory peak, which occurred 7 hours after induction. This finding is consistent with the pathophysiology of GA in humans^(32)^and the experimental model,^(12,14)^ with sudden onset and the pain peaked approximately 6-12 hours after induction.^(14)^ Moreover, pain is one of the primary clinical signs of the disease.^(10,32)^ The results of these experimental studies corroborate our findings. Tavares et al.^(14)^ induced GA with crystals in a rat model and observed an inflammatory peak 7 hours after induction. In another study using a murine model, a decrease in the nociceptive threshold was observed 6 hours later, and this threshold remained reduced for up to 15 hours, returning to baseline after 24 hours.^(12,13)^

Furthermore, we observed that the reduction in the nociceptive threshold in the arthritis group remained constant during all evaluations, suggesting that the inflammatory process induced by MSU crystals persisted, at least for the duration of the evaluations. Monosodium urate can induce the release of a series of inflammatory chemical mediators, such as substance P, serotonin, nitric oxide, and histamine, resulting in the accumulation of products from arachidonic acid metabolic pathways.^(33)^ In addition to the production of prostaglandins and leukotrienes, which directly activates nerve fibers, type Aδ and C, leading to changes in the afferent transduction threshold, MSU contributes to peripheral sensitization, thus causing hyperalgesia.^(34)^

Mobilization reversed the decrease in the nociceptive threshold observed in animals in the GAM Group, suggesting that passive joint mobilization during the inflammatory peak causes an analgesic effect. Mobilization has been shown to be a safe and useful technique for treating acute inflammatory conditions in other experimental models, such as the administration of carrageenan,^(25)^ capsaicin,^(35)^ and acute postoperative pain.^(36)^ A 9-minutes mobilization time was used in these studies, and an analgesic effect was also noted.^(25,35,36)^

One possible explanation for this effect is that the manual technique can activate mechanoreceptor Aß fibers, which are faster than C fibers, thereby promoting competitive inhibition in the dorsal horn of the spinal cord, favoring the suppression of the painful stimulus for supra-medullary regions.^(34)^ Joint mobilization can activate the descending inhibitory system, mediated by serotonergic and noradrenergic receptors,^(35)^ as the spinal pharmacological block of these receptors inhibited the analgesic effect of joint mobilization in a model of nociception induced by the administration of capsaicin in the ankle joints of rats. However, reports have demonstrated the involvement of the opioid and adenosinergic systems^(36)^ in the hypoalgesic action of mobilization in mouse and rat models of acute postoperative pain in the ankle.

The animals in the arthritis group exhibited a decrease in grip strength during the evaluations. This finding was similar to that observed by Tavares et al.^(14)^ in an acute model of GA in rats and by Montilla-García et al.^(37)^ in a chronic model of rheumatoid arthritis induced by Complete Freund’s adjuvant (CFA). Grip strength is widely used to assess the functional capacity of individuals with rheumatological inflammation;^(38)^ consequently, this acute and chronic inflammatory response induces muscle strength loss and functional disability.^(14,39)^

A possible explanation for the grip strength deficit may be tactile hypersensitivity and pain, as immersion cryotherapy decreased nerve conduction and increased the nociceptive threshold, facilitating the restoration of strength in an acute model of experimental GA.^(14)^Another reason for decreased pain and increased strength was observed by Montilla-García et al.,^(37)^ who used analgesic drugs that contributed to increased grip strength in a chronic model of experimental rheumatoid arthritis.

Additionally, loss of strength is associated with excruciating pain and muscle atrophy due to disuse.^(38)^ In the present study, joint mobilization treatment reversed the decrease in grip strength. This restoration of strength can be explained by the action of the autonomic nervous system, with an increase in sympathetic tone after joint mobilization, as reported by Sterling et al.^(24)^ after latero-postero-anterior mobilization of the C5-C6 cervical spine. Despite the limitations of scientific evidence, it is believed that the oscillatory movements of mobilization stimulate the joint mechanoreceptors and sensory fibers, thereby alleviating painful symptoms.^(19)^ With this, the animals could move the inflamed pelvic limb more in this 1-h period, preventing the consequences of disuse.^(26)^

It is worth mentioning that patients with GA have functional impairment with loss of muscle strength in their lower limbs^(38)^ and, in more severe and chronic cases, difficulty walking or reduced speed and cadence during gait,^(39)^ resulting in a lower quality of life when compared to individuals without the disease.^(40)^ The most severe impairment of muscle strength occurs during the tophaceous phase of the GA, where tophi obstruct tendon and muscle traction, reducing the cross-section and impairing the potential to generate strength.^(38)^ In addition, the proliferation of fibrotic tissue and collagen bridges causes adhesion and tension in the shortened muscle.^(41)^ Data observed in the research by Montilla-García et al.,^(37)^ which involved inducing rheumatoid arthritis in mice using CFA, showed a significant and prolonged reduction in the grip strength after induction. Therefore, our findings, although observed in the acute phase, suggest that joint mobilization at the inflammatory peak may contribute to the avoidance of functional limitations and disease progression.

Regarding the analysis of joint edema, the arthritic groups showed an increase in joint diameter and leukocyte cells, indicating that MSU crystals induced a local inflammatory process.^(10,14)^ This result was expected and has been observed in other experimental studies with induction of acute GA by crystals in mice^(12,13)^ and rats.^(9,14)^ The presence of crystals activates components of the innate immune system^(1)^ and stimulates the action of chemical mediators, such as histamine, bradykinin, leukotrienes, substance P, and nitric oxide, which promote hemodynamic changes with increased vascular permeability and vasodilation,^(34)^ favoring leukocyte efflux to the inflamed site and the development of inflammatory edema.^(12)^

Another mechanism that may have contributed to chemotaxis and the presence of inflammatory edema is the involvement of intraarticular crystals in the formation and activation of the NRLP3 inflammasome, with the activation of the myeloid differentiation primary response 88 (MYD88) protein that culminates in the release of interleukin-1 beta (IL-1ß) in humans^(42)^ and animals.^(13)^ Inhibition of IL-1ß and non-activation of the MYD88 protein has suggested a decrease in the inflammatory response in an acute GA model in mice.^(13)^ Furthermore, inhibition of IL-1ß appears to attenuate the release of several inflammatory cytokines, interfering in the regulation of chemokines such as chemokine C-X-C motif ligand 1 (CXCL1) through its receptors, chemokine C-C motif ligand 2 (CCL2) and chemokine C-C motif ligand 3 (CCL3), which can reduce chemotaxis and, consequently, edema and hyperalgesia in the ankle of mice in a GA-induced model.^(43)^

Additionally, there is evidence that interleukin-8 (IL-8) and CXCL1 bind to the IL-8 receptor beta (CXCR2) receptor to promote chemotaxis.^(44)^ Thus, cell migration appears to be mediated via activation of CXCR2 chemokine receptor, as this receptor significantly inhibits the recruitment of leukocytes in the knees of mice in an acute model of crystal-induced GA.^(13)^ Several aspects of these events favor leukocyte recruitment during an acute GA attack.^(42)^

Although leukocyte migration to the inflamed area was observed in the present study, there was no predominance of polymorphonuclear cells, unlike other experimental studies, such as in the acute model of gout in mice^(12,13)^ and rats,^(14)^ where a predominance of neutrophils was observed. The injection of MSU crystals induces local acute inflammation, where neutrophils are mobilized^(13)^ and gradually replaced by monocytes/macrophages, with attenuation of the influence of leukocyte infiltration in the synovial fluid. Thus, the presence of neutrophils is directly associated with the intensity of the inflammatory reaction.^(45)^

Treatment with joint mobilization reversed the increase in joint diameter immediately after treatment, and this effect persisted for at least the duration of the assessments. It is hypothesized that the mobilization technique acted directly on the connective tissue,^(20)^favoring pressure fluctuations within the joint cavity, which may have promoted the drainage of metabolites of the inflammatory process, such as arachidonic acid,^(33)^ which is directly involved in leukocyte migration and the inflammatory response.^(45)^ Therefore, it is believed that the oscillatory mobilization maneuver induced an immediate reduction in joint diameter, promoting edema reduction.^(41,45)^

Our findings suggest that mobilization may modulate the inflammatory process, as evidenced by the reduction in edema and subtle attenuation of cell migration, contributing to strength gain. Mobilization did not reverse leukocyte migration, a result that was also observed by Tavares et al.^(14)^ who induced GA in rats and performed a protocol with two sessions of cryotherapy by immersion, noting changes in the composition of the synovial fluid, a decrease in the percentage of monoarticular cells, and an increase in polymorphonuclear cell count. Cryotherapy reduced edema; however, the reduction was not persistent and did not reverse leukocyte migration. In contrast, Gomes et al.^(46)^ observed a decrease in nociception and edema and attenuation of leukocyte migration with a walking exercise protocol in a model of joint inflammation involving the administration of CFA in rats.

One limitation of this study was the inadequate volume of biological samples for molecular and biochemical analyses, which is attributed to the lack of studies utilizing the GA model and describing the anti-inflammatory pathways related to the mobilization protocol. These analyses may provide insights into the action of mobilization in the inflammatory response. This limitation was also encountered by Rulhen et al.,^(47)^ who induced joint inflammation in rats via carrageenan administration, performed a mobilization protocol, and did not observe attenuation of the inflammatory process in functional tests, suggesting that the effect of passive joint mobilization can be seen only at the molecular level or by other biochemical markers.

## CONCLUSION

The experimental model utilized in this study mimicked the characteristic signs of gouty arthritis during the inflammatory peak, such as decreased nociception threshold and grip strength, increased joint diameter, and elevated total leukocyte count. The protocol used for passive joint mobilization significantly increased the threshold of nociception and grip strength and reduced edema but did not reverse the increase in leukocytes. Therefore, manual therapy through joint mobilization improved functional parameters but did not demonstrate anti-inflammatory properties.
